# Effectiveness of cardiopulmonary bypass for radical resection of giant middle mediastinal paraganglioma

**DOI:** 10.34172/jcvtr.32907

**Published:** 2024-06-25

**Authors:** Nicola Rotolo, Andrea Imperatori, Luca Filipponi, Federica Torchio, Matteo Matteucci, Andrea Musazzi

**Affiliations:** ^1^Research Center of Minimally Invasive Surgery and Thoracic Surgery, Department of Medicine and Technological Innovation, University of Insubria, Varese, Italy; ^2^Research Center of Thoracic Surgery, Department of Medicine and Surgery, University of Insubria, Varese, Italy; ^3^Unit of Cardiac Surgery, ASST Settelaghi, University of Insubria, Varese, Italy

**Keywords:** Mediastinal paraganglioma, Cardiopulmonary bypass, Mediastinal tumors

## Abstract

A non-functional middle mediastinal paraganglioma is a rare entity. We describe a case of a 67-year-old woman with a diagnosis of a big mediastinal paraganglioma by endobronchial ultrasound transbronchial needle aspiration after chest CT and 18F-fluorodeoxyglucose positron-emission tomography. The nine centimeter in length tumor was located between the superior vena cava and the posterior portion of the ascending aorta, compressing the left atrium and the trachea and main left bronchus, posteriorly, surrounding the right pulmonary artery. Uniportal right video-thoracoscopic biopsy was unconclusive and complicated by severe hemorrhage, however controlled. Surgical resection was performed via a trans-sternal trans-pericardial approach followed by cardiopulmonary bypass and ascending aorta resection which allows an excellent exposure and greater control of great vessels and heart. Complete resection of the tumor was achieved without perioperative complication except for the left vocal cord palsy. Twelve months late the patient is disease free and in good general conditions.

## Introduction

 Mediastinal paragangliomas (MP) are a rare, usually asymptomatic neuroendocrine tumors considered to be of low-grade malignancy originating from the chromoaffin cell of extra adrenal paraganglia, accounting for 1% - 2% of all paraganglioma (0.3% of all mediastinal tumors).^1,2^ MP are often nonfunctional, can grow slowly and become large with surrounding compression and can also give metastasis. Often, is highly vascularized and located close to vascular structures (heart, great vessels, trachea, and main bronchus). Radical resection is the treatment of choice of MP; however, the radical surgery is a challenge and require a cardiopulmonary bypass (CPB) due to tenacious relationship with surrounding vascular structures, to prevent or control severe hemorrhages.

## Description of the case

**Figure 1:**
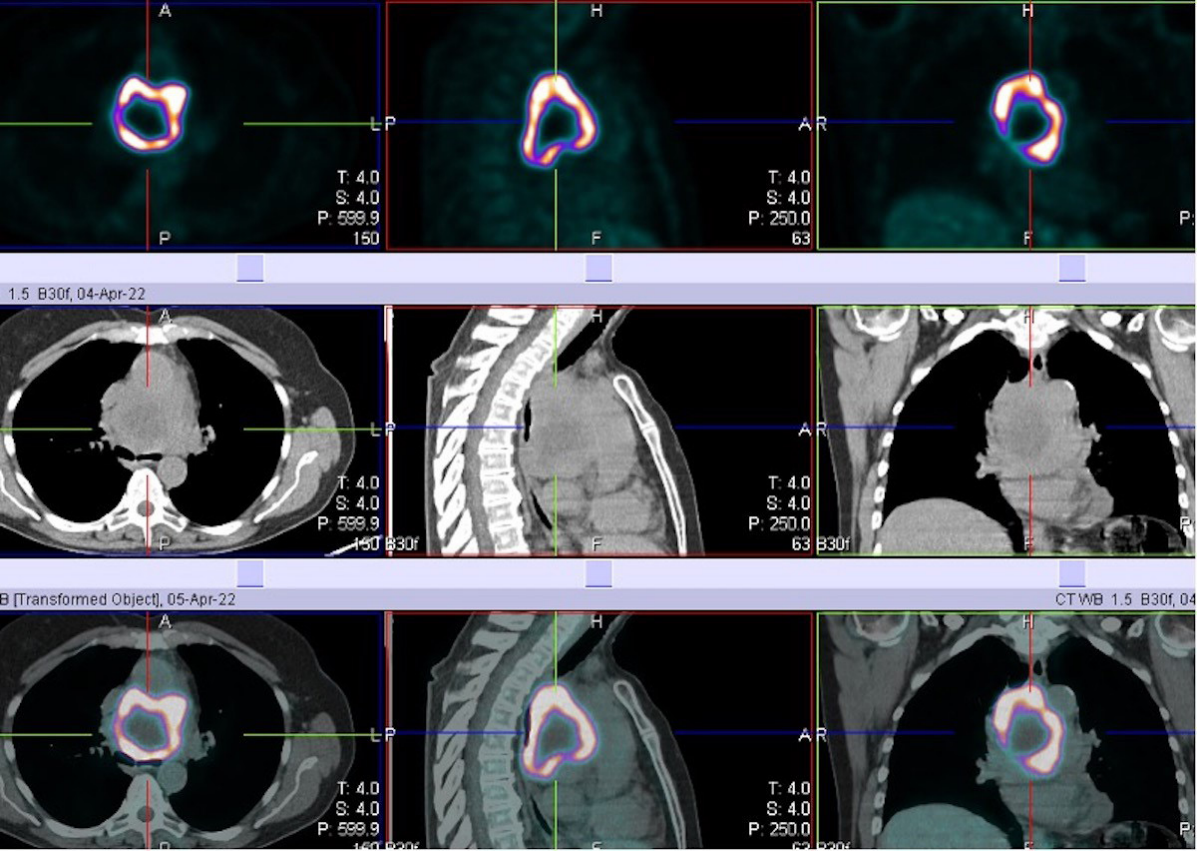
Positron emission tomography combined with computed tomography scan (PET-CT) showing very intense uptake (SUV 20) at middle mediastinum of 9 cm in diameter mass with central no metabolic fluorodeoxyglucose (FDG) activity

**Figure 2:**
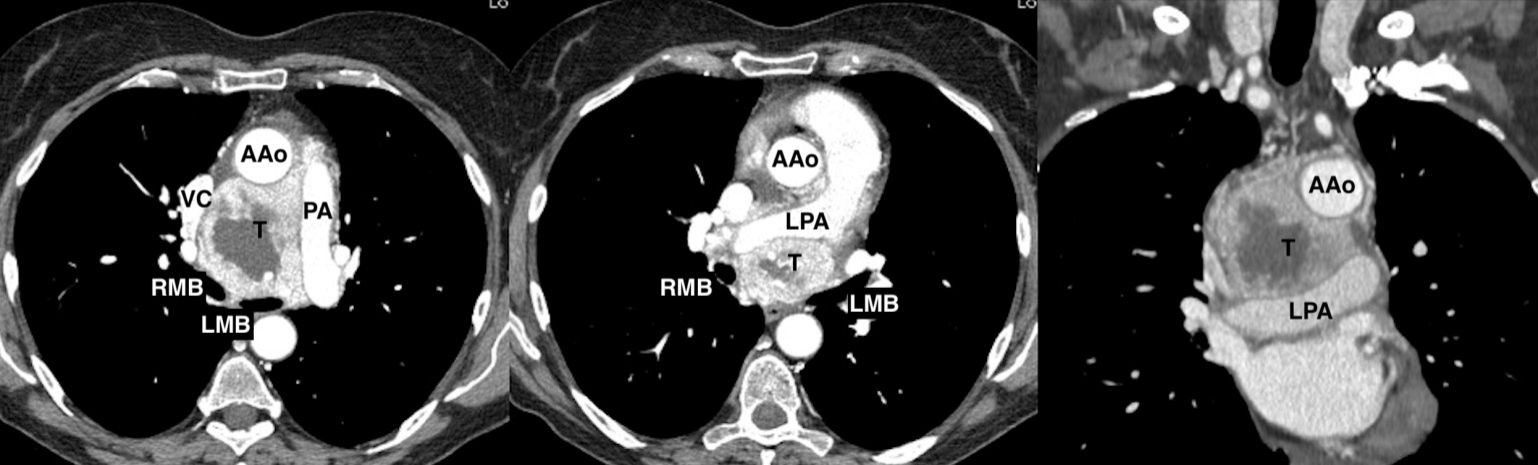
Axial and coronal enhanced chest computed scan (CTscan) showing a solid, highly peripherally vascularized, 7x6x3 cm mass in the middle mediastinum with low-density contrast enhancement in the central area; the tumor (T) was adjacent to the ascending aorta (AAo), superior vena cava (VC), atrium, surrounded the left pulmonary artery (LPA) and was tenaciously sticking to the trachea and the main bronchi (LMB, RMB). PA: Pulmonary artery

**Figure 3:**
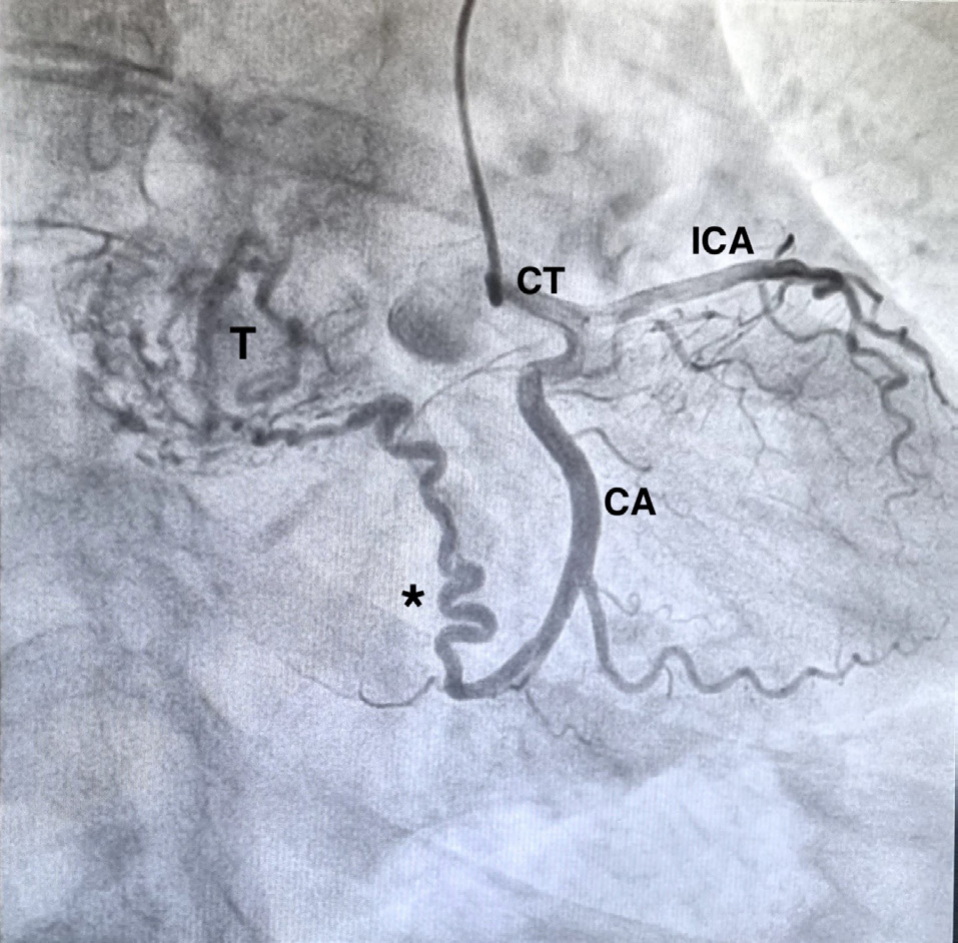
Coronarography revealed that the tumor (T) was perfused by a large tortuous vessel (asterisk) originating from the circumflex artery (CA). CT = Common trunk

 We report a case of a 67-year-old female who was incidentally diagnosed with a middle indeterminate mediastinal mass. Her past clinical history was characterized byarthritic symptoms as joint pain, loss of motion and inflammation. During follow-up, a Positron Emission Tomography (PET-scan) revealed a 9-cm middle mediastinal mass with a peripheral standardized uptake value (SUV = 20) with no evidence of extrathoracic uptake (Figure 1). A chest CT-scan revealed a large 70x60x30 mm well enhanced mass with necrotic central areas, located between the superior vena cava and the posterior portion of the ascending aorta, compressing the left atrium and, the trachea and main left bronchus posteriorly, surrounding the right pulmonary artery (Figure 2). A right uniportal video-thoracoscopic (U-VATS) biopsy of lesion was performed with a severe but controlled intraoperative bleeding. However, the mass remained undiagnosed so that a followed endobronchial-ultrasound transbronchial needle-aspiration (EBUS-TBNA), was suggestive of a paraganglioma. The patient was referred to surgery after multidisciplinary tumor board. A pre-operative coronarography revealed that the mass was perfused by a large tortuous vessel originating from the circumflex artery and smaller ones from the right coronary artery (Figure 3).

**Figure 4:**
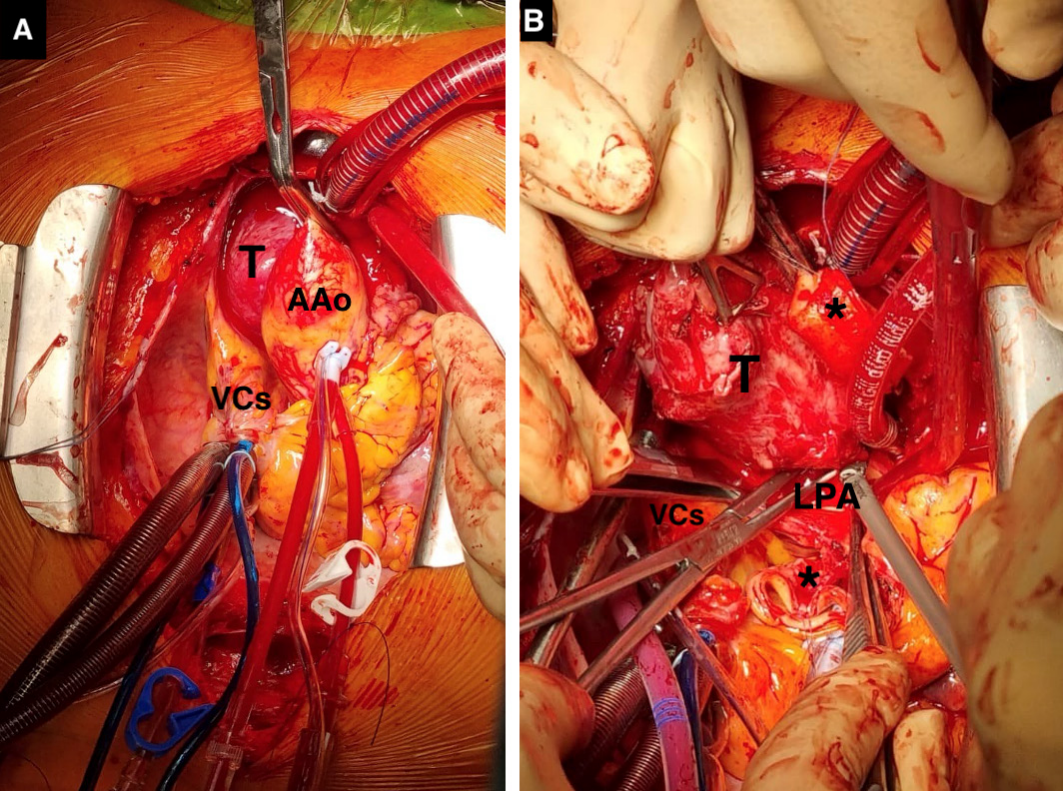
Intraoperative view via trans sternal trans pericardial sternotomy. 4A: Tumor (T) located between the aorta, the superior vena cava (VC), densely adherent to the posterior ascending aorta (AAo), the anterior wall of the trachea and to right pulmonary artery. 4B: Complete transection of the aorta (asterisks) after cardiopulmonary bypass, was necessary to achieve optimal exposure and complete removal of the tumor (T) surrounding left pulmonary artery (LPA)

 Via a trans-sternal trans-pericardial access, we identified a highly vascularized mass located between the aorta, the superior vena cava, the right atrium densely adherent to the posterior ascending aorta, the anterior wall of the trachea (until main left bronchus) and to the right pulmonary artery. Due to these tenacious adhesions and its rich blood supply CPB with bicaval cannulation was established, followed by aortic cross-clamping and cardiac arrest with blood cardioplegia. A complete transection of the aorta was necessary to achieve optimal exposure and complete removal of the tumor (Figure 4). CPB and aortic cross-clamping times were 172 and 143 min, respectively. The huge tumor was completely excised using ultrasonic dissector largelytoreduces bleeding and Metzenbaun scissors via blunt dissection; all small arteries and veins connected with the tumor were controlled by titanium clips. There is no evidence of local invasion into surrounding structures. The patient receiveda strict postoperative care in Intensive Care Unit and post-surgery course was uneventful, except for a left vocal cord paralysis.The patient was discharged on the 13th post-operative day.

 Histopathological examination revealed a solid, poly-lobulated, capsulated masswith central necrotic and hemorrhagic area, without extracapsular extension or vascular invasion, completely resected. Immunohistochemistry was positive for chromogranin, synaptophysin, and protein S-100 as low Ki67 proliferation index (5%) in addition to loss of succinate dehydrogenase complex iron-sulfur subunit B (SDHB) expression. The patient is alive without recurrence of disease 12 months after surgery.

## Discussion

 MP are a rare and highly vascularized tumors often located close to the vascular structures as heart, great vessels likewise trachea and main bronchus arising from the sympathetic ganglia of the great vessels.

 Complete resection of tumor is very difficult but mandatory for minimize local recurrences and metachronous metastasis occurring in 56% e 27 % of cases, respectively.^3^ The diagnosis of MP is incidentally or to begin with unspecific symptoms such as unexplained hypertensions, palpitations or like pheochromocytomas symptoms because of their production of norepinephrine and normetanephrine.^4^ In our case the diagnosis was made through a PET-CT scan performed for uncontrolled and persistent rheumatic symptom sunresponsive to medical treatment. FDG PET-CT is an important tool for evaluating polymyalgia rheumatica to diagnosis, therapeutic management, and follow-up of patients with arthritis, rheumatic disease and polymyalgia rheumatica as our patient. The use of PET-CT for evaluation of inflammatory conditions is increasingly used (X).

 Chest CT-scan, Magnetic Resonance Imaging, PET-scan and echocardiography are used to characterize the tumor size, location, and the relationship with surrounding structures. For the evaluation of mediastinal masses, the role of PET-CT may be complementary to chest CT-scan that provide detailed information about location, size, morphology, and extent of tumors. In cases of nonavid FDG lesion, PET-SCAN may reduce unnecessary invasive procedures for preoperative diagnosis. Even though a high metabolic activity of lesions may help to define its high probability of malignancy, however confirmatory tissue sampling is required to define the final diagnosis and to plan the right treatment. Radiologic imagines show strong and homogeneous peripheral enhancement and anecrotic central area that enhanced poorly. PET-scan confirms intense peripheral uptake due to rich blood supply and is useful for staging and follow-up. In addition, coronarography may be useful to understand possible blood supply that could cause accidental intraoperative blood loss. However, the pathological preoperative diagnosis is difficult because of the proximity of the tumor to the great vessels and its abundant blood supply. In our case, we performed an undiagnostic right U-VATS biopsy, complicated by a hemorrhage however resolved and, a few days later, we approached the tumor with an EBUS-TBNA under conscious sedation, using a 19-Gauche needle, successfully, nevertheless fine needle aspiration may be potentially misleading with a low diagnostic accuracy.^5^

 Surgical resection remains the cornerstone of management of MP depending on tumor localization and relationships with nearby structures. The sternotomy is the most used access^4^ often with CPB to facilitatetumor’s dissection mainly if located at the root of the ascending aorta or the main pulmonary artery, as in our case.^6^ In a series of 18 cases described by Liu, 56% of tumor was located at root of the ascending aorta and each one was resected with the help of cardiopulmonary bypass.^7^ Guerreri et al reported 10 of 22 resected cases undergoing cardiopulmonary bypass for tumor’s invasion of atrioventricular groove, right and left atrium, distal descending aorta, and arch.^8^

 Complete resection of the tumor can have an extremely high risk, with 6% of the mortality (reported range: 3.5% -20%),^9^ mainly due to massive hemorrhagethat is the main cause of intraoperative death. The adoption of the CPB can create favorable conditions for en-bloc resection ensuring hemodynamic and respiratory stability.^10^ The use of the harmonic scalpel to dissect the tumor was associated with significant reduction of intra and post operative bleeding.^11^ To minimize bleeding risk during surgical excision of the tumor, some authors prefer to embolize the mass afferent arteries.^12^ However, preoperative embolization does not reduce the risk of surrounded vessels lesion like pulmonary artery or upper vena cava; in this cases, the CPB reduce the pressure on the great vessels making removal the tumor with more confidence, and in cases of severe invasion of vascular structures their reconstruction/replacement may be necessary.^4^ If bleeding occurs, it’s difficult to assure a clear operative field but above all hemodynamic stability without cardiopulmonary bypass. However, the prompt institution in emergency conditions can be lifesaving.

 Complete surgical resection remains the standard of care associated with excellent survival: the 5- and 10-year survival rates were 88% and 78%, respectively as reported in a series by Hamidi.^13^ Radiation and chemotherapy appear to be ineffective so what radical resection is an important prognostic factor.^14^

 Life-long surveillance for local recurrence and metastatic spread is mandatory; due to their own metabolic avidity, the PET-Scan is auseful half-yearly method of follow-up investigation.

 Up to 25% of paraganglioma neoplasms are hereditary; genetic tests should be considered in all patients.^15^ They detect specific germline mutations SDHB related to a greater biological aggression.^16^ Our patient’s genetic SDH test was negative.

## Conclusion

 Resection of MP is a challenge and high-risk procedure for potential uncontrolled, catastrophic hemorrhage. However, complete surgical resection is the treatment of choice related to an excellent survival. A careful preoperative evaluation and a multidisciplinary approach are required for all mediastinal localization. The CPB is an effectivetool for patient safety and oncological surgical radicality.

## Competing Interests

 The authors declare that they have no competing interests.

## Ethical Approval

 As case report, no ethical approval was required. The patient signed the written informed consent for the publication of this case report study including all the images, available for reviewers. The patient was assured that all informations will remain anonymous and confidential.
